# Long term clinical outcomes of minimally invasive transforaminal interbody fusion (MIS-TLIF) for lumbar spondylolisthesis in a geriatric (>65 years) population: a systematic review and meta-analysis

**DOI:** 10.3389/fsurg.2025.1517947

**Published:** 2025-03-21

**Authors:** Talgat Kerimbayev, Yerzhan Kuanyshbekov, Serik Akshulakov, Indira Karibayeva

**Affiliations:** ^1^ Department of Spinal Neurosurgery and Peripheral Nervous System Pathology, National Center for Neurosurgery, Astana, Kazakhstan; ^2^ Department of Neurosurgery, National Center for Neurosurgery, Astana, Kazakhstan; ^3^ Jiann-Ping Hsu College of Public Health, Georgia Southern University, Statesboro, GA, United States

**Keywords:** MIS-TLIF, geriatric patients, lumbar spondylolisthesis, systematic review, meta-analysis

## Abstract

**Introduction:**

The minimally invasive transforaminal lumbar interbody fusion (MIS-TLIF) technique has become a popular and effective option for treating lumbar degenerative spondylolisthesis, especially in elderly patients. This systematic review and meta-analysis is to evaluate the long-term results of MIS-TLIF for patients with degenerative spondylolisthesis.

**Methods:**

We thoroughly reviewed and analyzed studies from databases like PubMed, Web of Science, Scopus, and Google Scholar, covering research published from 2015–2024. We used random-effects models to estimate overall prevalence, and we conducted sensitivity analyses and assessed publication bias to understand the variability in results. All analyses were done using the “meta” and “metafor” packages in RStudio.

**Results:**

According to the random-effects model, the pooled standardized mean difference of the VAS back score dynamics at 12 months post-operative in geriatric MIS-TLIF patients was −4.30, 95% CI [−10.02; 1.42]; the VAS leg pain score dynamics at 12 months post-operative was −2.46, 95% CI [−5.61; 0.68]; the ODI score dynamics at 12 months post-operative was −3.01, 95% CI [−6.02; −0.01]. The VAS back pain score dynamics at 24 months post-operative was −1.77, 95% CI [−2.33; −1.21]; the VAS leg pain score dynamics at 24 months post-operative was −2.29, 95% CI [−3.22; −1.37]; and the ODI score dynamics at 24 months post-operative was −1.92, 95% CI [−2.57; −1.27].

**Conclusion:**

Our study provides compelling evidence supporting the long-term efficacy of MIS-TLIF for managing lumbar spondylolisthesis in geriatric patients. The findings suggest that MIS-TLIF is associated with significant reductions in back and leg pain, as well as improvements in disability scores over 12 months post-operatively. However, these improvements in pain and functional disability decline at 24 months postoperatively, which could be explained by the physiological nature of degenerative changes in the geriatric population.

**Systematic Review Registration:**

https://www.crd.york.ac.uk/PROSPERO/view/CRD42024538220, PROSPERO (CRD42024538220).

## Introduction

Lumbar spondylolisthesis—a common spinal disease in which one vertebra slips forwards over the vertebra below—serious impairment to the heath and quality of life in the elderly (1). Reports suggest that approximately one in five adults may experience spondylolisthesis, with certain variations being significantly more prevalent in older populations (2). Symptoms often include chronic lower back pain, radiculopathy, and neurogenic claudication, resulting in the loss of mobility and functionality (3). This can affect their physical well-being and contribute to their loss of independence, social withdrawal, and depression (4). Besides, lumbar spondylolisthesis could affect all aspects of health-related quality of life as assessed using the Short-Form Health Survey (SF-36) (5). The impact of lumbar spondylolisthesis in elderly patients brings to focus the need for proper management to ensure better outcomes and enhance the well-being of the patients affected (6).

The global population is experiencing a significant aging trend, with a continuous increase in the number of individuals aged 65 and older. Among patients who underwent geriatric neurosurgery, the mortality rate was 6.5 percent (7).Seventeen percent of the total diagnoses and eleven percent of diagnoses in patients over 85 years of age were degenerative myeloradiculopathy of the spine. In elderly individuals, degenerative processes in the spine often lead to severe, intolerable pain, with lumbar spondylolisthesis occurring particularly frequently, affecting 72 percent of such patients (7).

In recent years, minimally invasive transforaminal lumbar interbody fusion (MIS-TLIF) has been progressively applied in elderly patients with lumbar degenerative diseases (8). MIS-TLIF is an ideal combination of radicular decompression and spondylodesis, offering a minimally invasive approach (9). Moreover, MIS-TLIF has various benefits over open surgery, including reductions in blood loss, tissue trauma and postoperative pain, and is associated with earlier mobilization from bed and shorter hospital stays (9). However, there are still limited studies exploring its specific indications and clinical outcomes in the elderly population.

A recent systematic review and meta-analysis focusing on short-term (6 months) outcomes and complications of MIS-TLIF found notable improvements in the Visual Analogue Scale (VAS) back pain score, the VAS leg pain score, and the Oswestry Disability Index (ODI) with cumulative mean differences of −3.87 [95% CI (−4.97; −2.77)], −5.11 [95% CI (−6.69; −3.53)], and −30.70 [95% CI (−41.84; −19.55)], respectively (10). Previous studies have indicated a higher incidence of reoperations and complications in geriatric patients undergoing spine surgery (11).

To the best of the authors' knowledge, no study has yet examined the long-term clinical outcomes of 69 MIS-TLIF surgery for lumbar spondylolisthesis in the geriatric population. Therefore, this study aims to systematically review the existing literature and conduct a meta-analysis to thoroughly investigate the long-term postoperative outcomes (12 and 24 months) for geriatric individuals who have undergone MIS-TLIF surgery.

## Materials and methods

The study protocol is registered with the PROSPERO International prospective register of systematic reviews. (Reference: CRD42024538220).

### Search strategy

The PROSPERO database was searched to identify the registration of similar studies, but no similar studies were found. We conducted a subsequent search in four major electronic literature databases: PubMed, Web of Science, Scopus, and Google Scholar. The literature search in the specified sources was initiated on January 1, 2024, and completed on March 1, 2024. The search strategy included the following keywords: “minimally invasive and spondylolisthesis”; “MIS-TLIF and spondylolisthesis”; “fusion and spondylolisthesis”; “minimally invasive transforaminal interbody fusion and elderly”; and “Geriatric and MIS-TLIF”. The search results were restricted to publications from the year 2015. The full strategy is presented in supplementary materials (Supplementary Table S1).

### Eligibility criteria

Methodologically, the literature screening and synthesis adhered to the guidelines outlined in the Preferred Reporting Items for Systematic Reviews and Meta-Analyses (PRISMA) (12). The inclusion criteria for this systematic review were defined using the PICOS (Population, Intervention, Comparison, Outcome, Study Design) framework as follows: Population (P): Studies focusing on geriatric patients aged 65 years and older diagnosed with lumbar spondylolisthesis. Intervention (I): Minimally Invasive Transforaminal Interbody Fusion (MIS-TLIF). Comparison (C): None. Outcomes (O): Studies reporting specific clinical outcomes, including Visual Analog Scale (VAS) scores for pain and Oswestry Disability Index (ODI) scores for functional disability, assessed at 12 and 24 months postoperatively. Study Design (S): Cohort studies, cross-sectional studies, randomized clinical trials (RCTs), and database analyses published in English. Conversely, exclusion criteria encompassed: (1) publications lacking essential information; (2) studies that duplicated the findings of articles already included in the reported analysis; (3) articles focusing on surgical interventions for geriatric patients with conditions such as disc herniation, scoliosis, fractures, or infection; (4) review articles or case reports involving fewer than ten patients.

### Selection of studies and data extraction

The identified publications underwent a rigorous process, including deduplication and primary (title + abstract) and eligibility (full text) reviews. Subsequently, comprehensive full-text perusal, review, and data extraction were conducted, with each stage resulting in the exclusion of publications based on predefined inclusion and exclusion criteria. Adhering to the PRISMA guidelines, two authors independently extracted pertinent information from the identified full-text articles using a standardized data extraction form. The data of interest encompassed various aspects such as the first author's name, publication year, country, study design, lesion location, mean age, VAS leg and VAS back pain scores, ODI scores, and assessment period. In instances of disagreement between authors, consensus was achieved through thorough discussion and consultation with a third author.

### Risk of bias

The Critical Appraisal Skills Programme (CASP) Checklist for Cohort studies was employed to evaluate the methodological quality of the included studies. This checklist comprised ten questions covering various aspects, such as the study's objectives, methodology, research design, recruitment approach, data collection methods, researcher-participant relationships, ethical considerations, data analysis, research findings, and overall value. Each criterion was assessed with a rating of “yes” when adequately described (scored as 1), “no” when absent (scored as 0), and “can't tell” when unclear or incomplete (scored as 0.5). The total scores ranged from 0–10, with a score of at least 7 considered indicative of satisfactory quality (Table 1).

**Table 1:** The risk of bias assessment results based on the critical appraisal skills programme checklist.

Author, year	Aim	Methodology	Design	Recruitment	Data collection	Relationship	Ethical	Data analysis	Finding	Values	Score
Goh, et al. (13)	YES	YES	YES	YES	YES	Can’t tell	YES	YES	YES	YES	9,50
Büyük, et al. (6)	YES	YES	YES	YES	YES	YES	YES	YES	YES	YES	10
Qin, et al. (14)	YES	YES	YES	YES	YES	YES	YES	Can’t tell	YES	YES	9,50
Goh, et al. (15)	YES	YES	YES	YES	YES	YES	YES	Can’t tell	YES	YES	9,50
Lin, et al. (16)	YES	YES	YES	YES	YES	YES	YES	YES	YES	YES	10
Lee, et al. (17)	YES	YES	YES	YES	YES	YES	YES	Can’t tell	YES	YES	9,50
Nikhil, et al. (18)	YES	YES	YES	YES	YES	YES	YES	YES	YES	YES	10

### Statistical analysis

We used RStudio to calculate the pooled standardized mean difference (SMD) with 95% confidence intervals. We did this using a random-effects model for meta-analysis. We looked at the following outcomes: VAS back pain at 12 and 24 months, VAS leg pain at 12 and 24 months, and ODI at 12 and 24 months. Forest plots were used to display the pooled estimates using the “RevMan5” layout function. Heterogeneity across studies was assessed using the IZ-statistic. Publication bias was evaluated through visual inspection of a funnel plot and statistical analysis using Egger's test, examining potential asymmetry in the distribution of study results.

## Results

**Figure 1:**
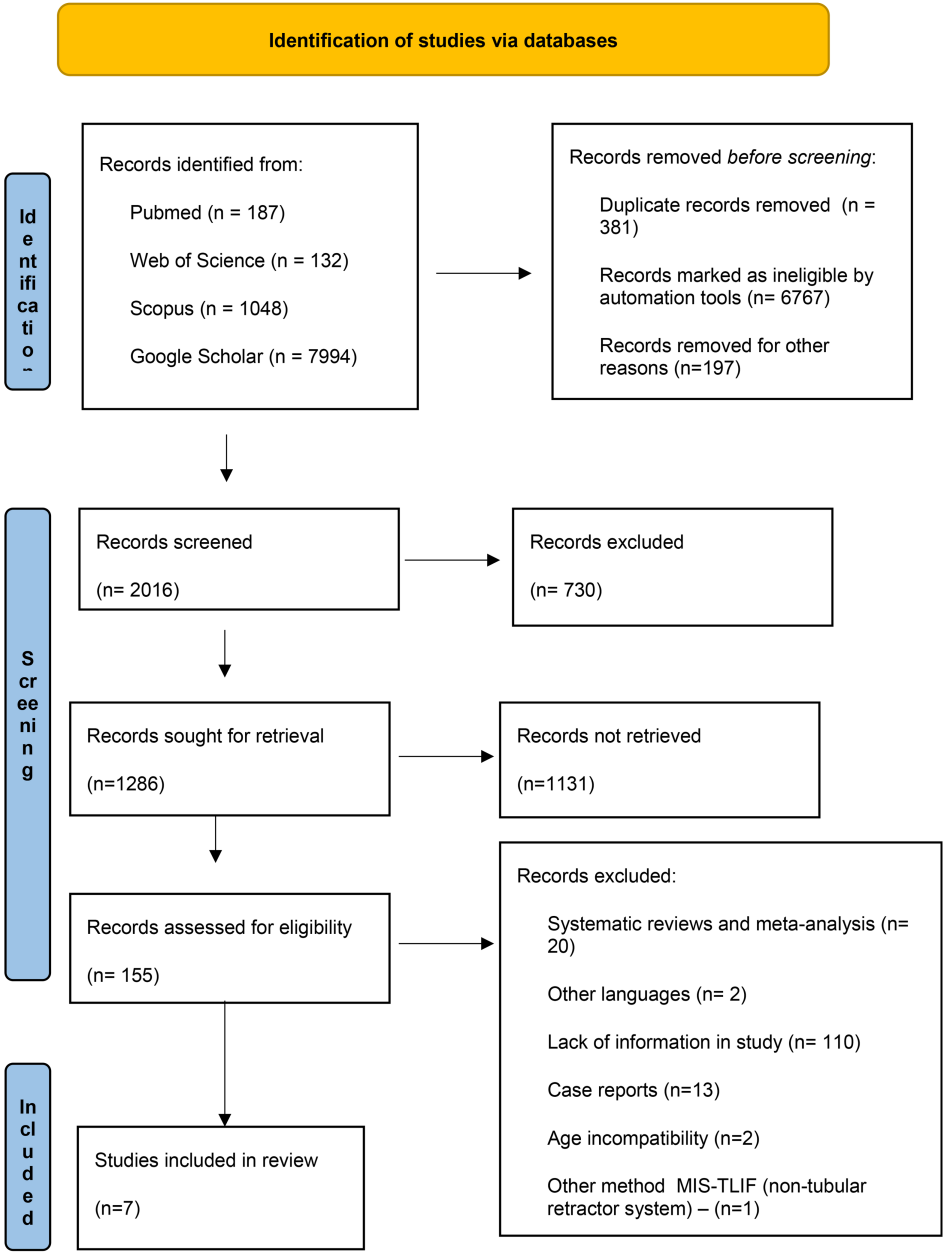
PRISMA flow chart of study selection.

A thorough search across PubMed, Web of Science, Scopus and Google Scholar databases yielded 9,361 records. Initial screening reduced this to 8,980 non-duplicative records, from which 155 full-text articles underwent evaluation. Of these, 7 studies met the inclusion and exclusion criteria, and were included in this systematic review. We excluded 3 studies that reported mean VAS scores for back and leg pain, as well as ODI scores, but did not provide standard deviations [Hyeong-Jin Lee (19), Won-Suh Choi (20), Pei-I Hung (21)]. One additional study was excluded because it used a different MIS-TLIF method involving a non-tubular retractor system (Myeong Jin Ko (22). Furthermore, 2 articles were excluded due to age category constraints [Andrew K. Chan (23), Kenyu Ito (24)]. The study selection process is detailed in Figure 1.

### Description of included studies and subjects

The study design and patient characteristics are delineated in Table 2. Notably, all included studies were published within the timeframe of 2015–2024, underscoring the contemporary relevance of the subject matter. Geographically, the studies exhibited diverse origins: three emanated from East Asia, three from Southeast Asia and one from Western Asia (Table 2).

**Table 2:** Description of the included articles.

First author, year	Country/WHO region	Study design	Patient number	Mean age of study group ± SD or age groups	Mean follow up	Procedure type	Outcome measures	Surgical tecnique
Goh, et al. (13)	Singapore/Southeast Asia	Retrospective cohort study	30	73.5 ± 3.4	7.2 ± 2.0 Years	MIS-TLIF	VAS back, VAS leg, ODI score.	Tubular retractor system
Büyük, et al. (6)	Turkey/Western Asia	Retrospective cohort study	45	69 (65–74)	2 years	MIS-TLIF	VAS back, VAS leg, ODI score.	Tubular retractor system
			23	77 (75–84)	2 years	MIS-TLIF	VAS back, VAS leg, ODI score.	Tubular retractor system
Qin, et al. (14)	China/East Asia	Retrospective cohort study	31	66.09 ± 8.19	2 years	MIS-TLIF	VAS back, ODI score.	Tubular retractor system
Goh, et al. (15)	Singapore/Southeast Asia	Retrospective cohort study	39	75 ± 3	3.9 ± 1.5 years	MIS-TLIF	VAS back, ODI score.	Tubular retractor system
Lin, et al. (16)	Korea/East Asia	Retrospective cohort study	41	72.27 ± 4.41	18.98 months	MIS-TLIF	VAS back, VAS leg, ODI score.	Tubular retractor system
Lee, et al. (17)	Korea/East Asia	Retrospective cohort study	27	60.55 ± 13.61	15.5 ± 11.61 1 year	MIS-TLIF	VAS back, ODI score.	Tubular retractor system
Nikhil, et al. (18)	Singapore/Southeast Asia	Retrospective cohort study	22	78.18 ± 2.58	49.41 ± 20.83 months	MIS-TLIF	VAS back, VAS leg, ODI	Tubular retractor system
			22	69.99 ± 2.56	49.77 ± 19.67 months	MIS-TLIF	VAS back, VAS leg, ODI score.	Tubular retractor system

### Subjects

A total of 280 patients with lumbar degenerative spondylolisthesis were included in ten studies (mean sample size = 40 patients, range = 22–45 patients). The mean age of the participants ranged from 60,55–78,18 years. The mean follow-up ranged from 1 year–7.2 years.

### The risk of bias assessment

The risk of bias assessment results are presented in Table 1. All studies had a low risk of bias, with four studies scoring 9.5, and three studies scoring 10 out of 10 possible points on the CASP scale.

## Post-Operative outcomes

### VAS back pain, leg pain, and ODI score at 12 months post-operative

**Figure 2:**
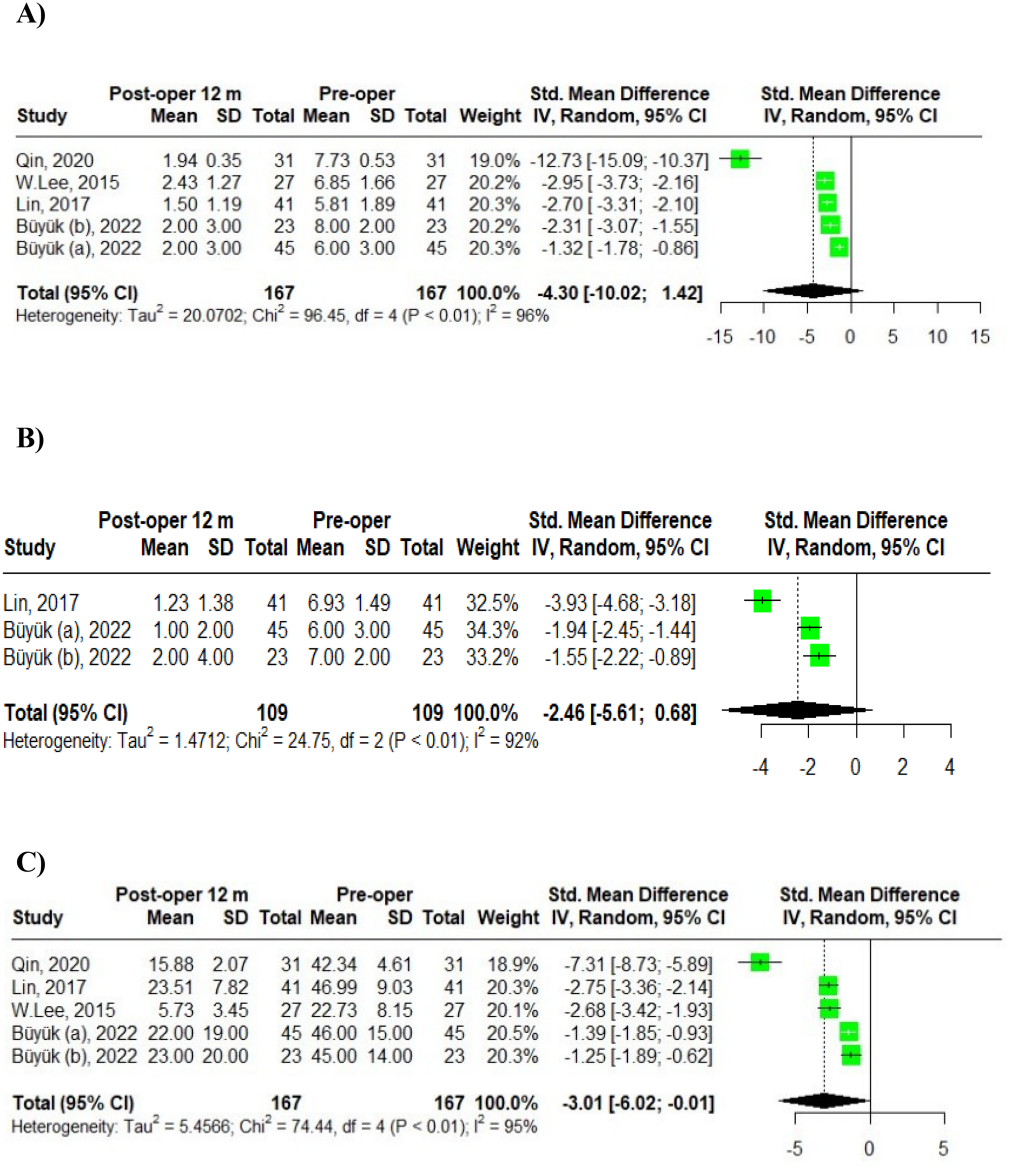
Meta-analysis of 12-month post-operative outcomes in MIS-TLIF patients: **(A)** VAS back pain; **(B)** VAS leg pain; **(C)** ODI scores.

Four studies comprising five groups reported the dynamics of VAS back pain scores at 12 months, postoperatively. Buyuk (6) categorized the VAS back pain scores into two age groups: scores for patients aged 65–74 were labeled as Buyuk (a), while those for patients aged 75–84 years were labelled as Buyuk (b). Using a random-effects model, the pooled standardized mean difference (SMD) for the VAS back pain score dynamics among the five groups of geriatric MIS-TLIF patients was −4.30, with a 95% confidence interval (CI) [−10.02; 1.42]. The heterogeneity test indicated a high degree of variability among the studies, with an IZ of 96%, a Q statistic of 96.45 (df = 4), and a *p*-value of <0.01 (Figure 2A).

Two studies involving three groups examined the dynamics of VAS leg pain scores at 12 months post-operation. Buyuk et al. (6) reported VAS leg pain scores categorized into two age groups: scores for patients aged 65–74 were labeled as Buyuk (a), and those for patients aged 75–84 years were labeled as Buyuk (b). Utilizing a random-effects model, the pooled standardized mean difference (SMD) for VAS leg pain score dynamics among the three groups of geriatric MIS-TLIF patients was −2.46, with a 95% confidence interval (CI) [−5.61; 0.68]. The heterogeneity test indicated a high degree of variability: IZ = 92%, *Q* (df = 2) = 24.75, *p*-value < 0.01 (Figure 2B).

Additionally, four studies involving five groups assessed the dynamics of ODI scores at 12 months post-operation. Again, Buyuk et al. (6) divided the ODI scores into two age groups with scores for patients aged 65–74 were presented as Buyuk (a), and those for patients aged 75–84 as Buyuk (b). The pooled SMD for ODI score dynamics among the five groups was −3.01, with a 95% CI of [−6.02; −0.01]. The heterogeneity analysis revealed significant variability: IZ = 95%, *Q* (df = 4) = 74.44, *p*-value < 0.01 (Figure 2C).

### Sensitivity analysis

Sensitivity analysis assessed the robustness of the pooled estimates. The results revealed persistent heterogeneity, and the pooled prevalence estimate for the VAS back pain score dynamics at 12-months post-operative did not remain stable G = −2.29, 95% CI [−3.02; −1.55], when the outlier was removed. The pooled prevalence estimate results for the VAS leg pain score dynamics at 12-months post-operative remained stable G = −2.46, 95% CI [−3.89; −1.04]. The results revealed persistent heterogeneity, and the pooled prevalence estimate for the ODI score dynamics at 12-month post- operative did not remain stable G = −2.00, 95% CI [−2.79; −1.21], when the outlier was removed. Outlier analysis results are presented in Table 3.

**Table 3:** Outlier-analysis of the VAS back pain, VAS leg pain, and ODI score dynamics at 12 months post-operative in TLIF patients.

Variable	Stability	*G* Value	95% CI
VAS back pain score	Not stable	−2.29.	(−3.02; −1.55)
VAS leg pain score	Stable	−2.46.	(−3.89; −1.04)
ODI score	Not stable	−2.00.	(−2.79; −1.21)

**Figure 3:**
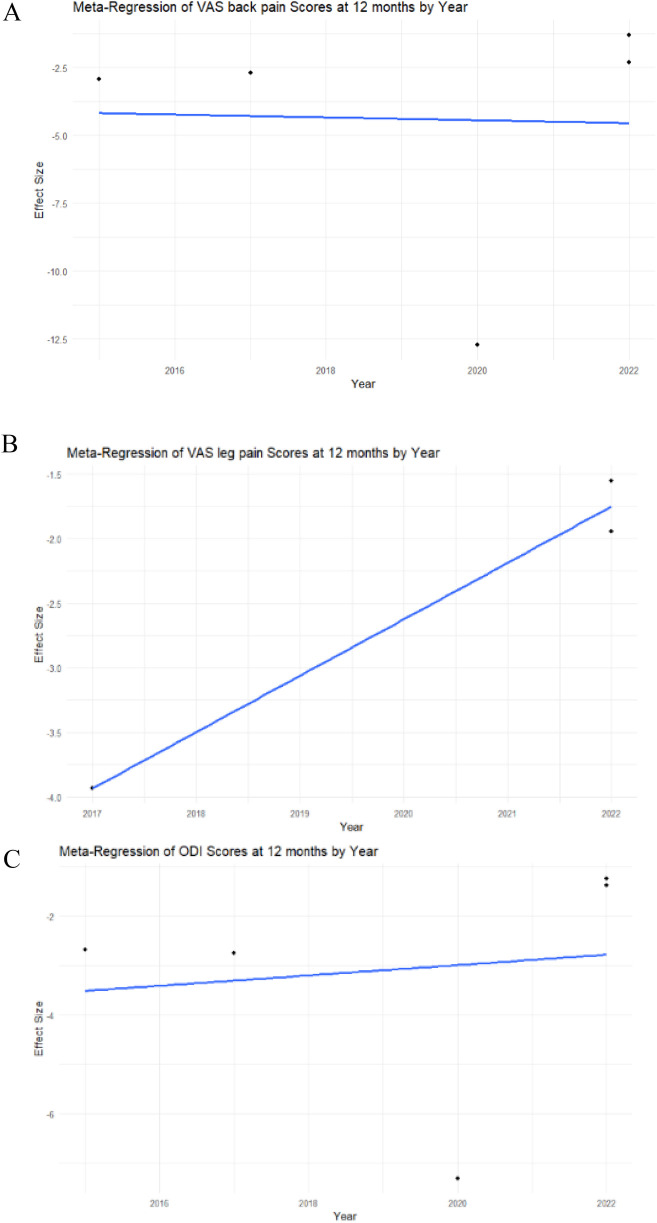
Meta-regression analysis of the VAS back and leg pain, ODI scores in MIS-TLIF patients. **(A)** VAS back pain score; **(B)** VAS leg pain score; **(C)** ODI score dynamics at 12 months post-operative in MIS-TLIF patients.

A meta-regression by year was performed for each study to assess heterogeneity. In the analysis of VAS back pain at 12 months, meta-regression showed statistical insignificance (intercept *p*-value = 0.9604), indicating that the examined factor did not have a significant impact on changes in the VAS back pain score in this model (Figure 3A).

The meta-regression analysis of VAS leg pain at 12 months demonstrated statistical significance (intercept *p*-value < 0.0001), suggesting that the examined factor significantly influenced changes in the VAS leg pain score in this model (Figure 3B).

The meta-regression analysis of ODI at 12 months demonstrated statistical insignificance (intercept *p*-value = 0.7985), indicating that the examined factor did not have a significant impact on changes in the ODI score in this model (Figure 3C).

### Publication bias assessment

**Figure 4:**
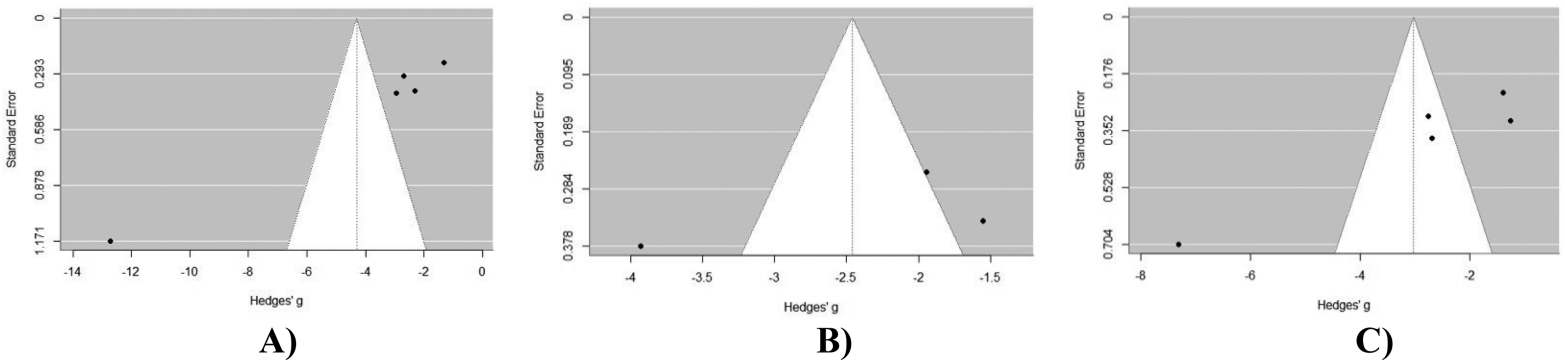
Publication bias assessment of outcomes at 12 months post-operatively in MIS-TLIF patients: **(A)** VAS back pain score; **(B)** VAS leg pain score; **(C)** ODI score dynamics at 12 months post-operative in MIS-TLIF patients.

Upon visual inspection of the VAS back pain funnel plot (Figure 4A), asymmetry is evident, suggesting a non-symmetric distribution of study results around the estimated effect size. This finding was further confirmed by Egger's test for publication bias, which yielded significant results (*p* < 0.05). Upon visual inspection of the VAS leg pain funnel plot (Figure 4B), asymmetry is not evident, suggesting a symmetric distribution of study results around the estimated effect size. This finding was further confirmed by Egger's test for publication bias, which yielded non-significant results (*p* > 0.05). Upon visual inspection of the ODI score funnel plot (Figure 4C), asymmetry is evident. This finding was further confirmed by Egger's test for publication bias, which yielded significant results (*p* < 0.05).

### VAS back pain, VAS leg pain, and ODI score at 24 months post-operative

**Figure 5:**
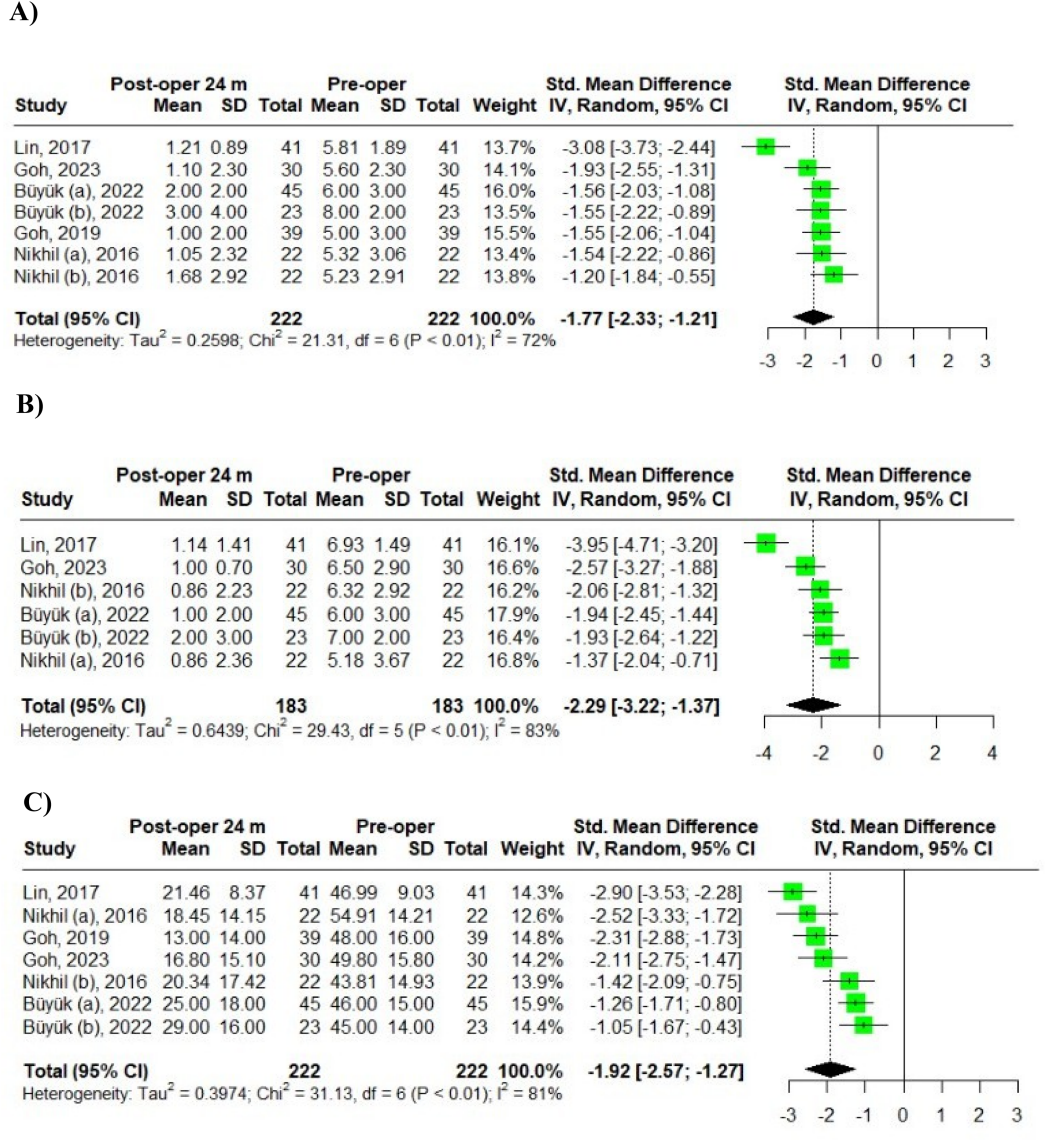
Meta-analysis of 24-month post-operative outcomes in MIS-TLIF patients: **(A)** VAS back pain; **(B)** VAS leg pain; **(C)** ODI scores.

Five studies with seven groups presented the dynamics of VAS back pain scores at 24 months postoperation. Buyuk et al. (6) divided the VAS back pain scores into two age groups: scores for patients aged 65–74 were labeled as Buyuk (a), while those for patients aged 75–84 years were labeled as Buyuk (b). Similarly, Nikhil et al. (18) categorized VAS back pain scores into two age groups: scores for the old-old group were designated as Nikhil (a), and those for the young-old group were marked as Nikhil (b). According to the random-effects model, the pooled standard mean difference (SMD) for the dynamics of VAS back pain scores among the seven groups was −1.77, with a 95% confidence interval (CI) of [−2.33, −1.21]. The heterogeneity test indicated high variability: IZ = 72%, *Q* (df = 6) = 21.31, *p*-value < 0.01 (Figure 5A).

Four studies with six groups assessed the dynamics of VAS leg pain scores at 24 months. Again, Buyuk et al. (6) divided the VAS leg pain scores into two age categories, with scores for patients aged 65–74 labeled as Buyuk (a), and those for patients aged 75–84 as Buyuk (b). Similarly, Nikhil et al. (18) categorized VAS leg pain scores into two age groups: scores for the old-old group were designated as Nikhil (a), while those for the young-old group were labeled as Nikhil (b). The random-effects model revealed a pooled SMD of −2.29 for the dynamics of VAS leg pain scores among the six groups, with a 95% CI of [−3.22, −1.37]. The heterogeneity analysis indicated high variability: IZ = 83%, *Q* (df = 5) = 29.43, *p*-value < 0.01 (Figure 5B).

Additionally, five studies with seven groups evaluated the dynamics of ODI scores at 24 months. Buyuk et al. (6) again divided ODI scores into two age groups: scores for patients aged 65–74 were labeled as Buyuk (a), and those for patients aged 75–84 as Buyuk (b). Likewise, Nikhil et al. (18) categorized ODI scores into two age groups: scores for the old-old group were designated as Nikhil (a), while those for the young-old group were labeled as Nikhil (b). Based on the random-effects model, the pooled SMD for the dynamics of ODI scores among the seven groups was −1.92, with a 95% CI of [−2.57, −1.27]. The heterogeneity analysis showed high variability: IZ = 81%, *Q* (df = 6) = 31.13, *p*-value < 0.01 (Figure 5C).

### Sensitivity analysis

Sensitivity analysis assessed the robustness of the pooled estimate. The pooled prevalence estimate for the VAS back pain score dynamics at 24-month post-operative did not remain stable, G = −1.56, 95% CI [−1.8;−1.32]. The results revealed persistent heterogeneity, and the pooled prevalence estimate for the VAS leg pain score dynamics at 24-month post-operative did not remain stable G = −1.97, 95% CI [−2.32; −1.61], when the outlier was removed. The pooled results prevalence estimate for the ODI score dynamics at 24-months post-operative remained stable G = −1.92, 95% CI [−2.45; −1.4]. Outlier analysis results are presented in Table 4.

**Table 4:** Outlier-analysis of the VAS back pain, VAS leg pain and ODI score dynamics at 24 months post-operative in TLIF patients.

Variable	Stability	*G* Value	95% CI
VAS back pain score	Not stable	−1.56	(−1.8; −1.32)
VAS leg pain score	Stable	−1.97	(−2.32; −1.61)
ODI score	Not stable	−1.92	(−2.45; −1.4)

**Figure 6:**
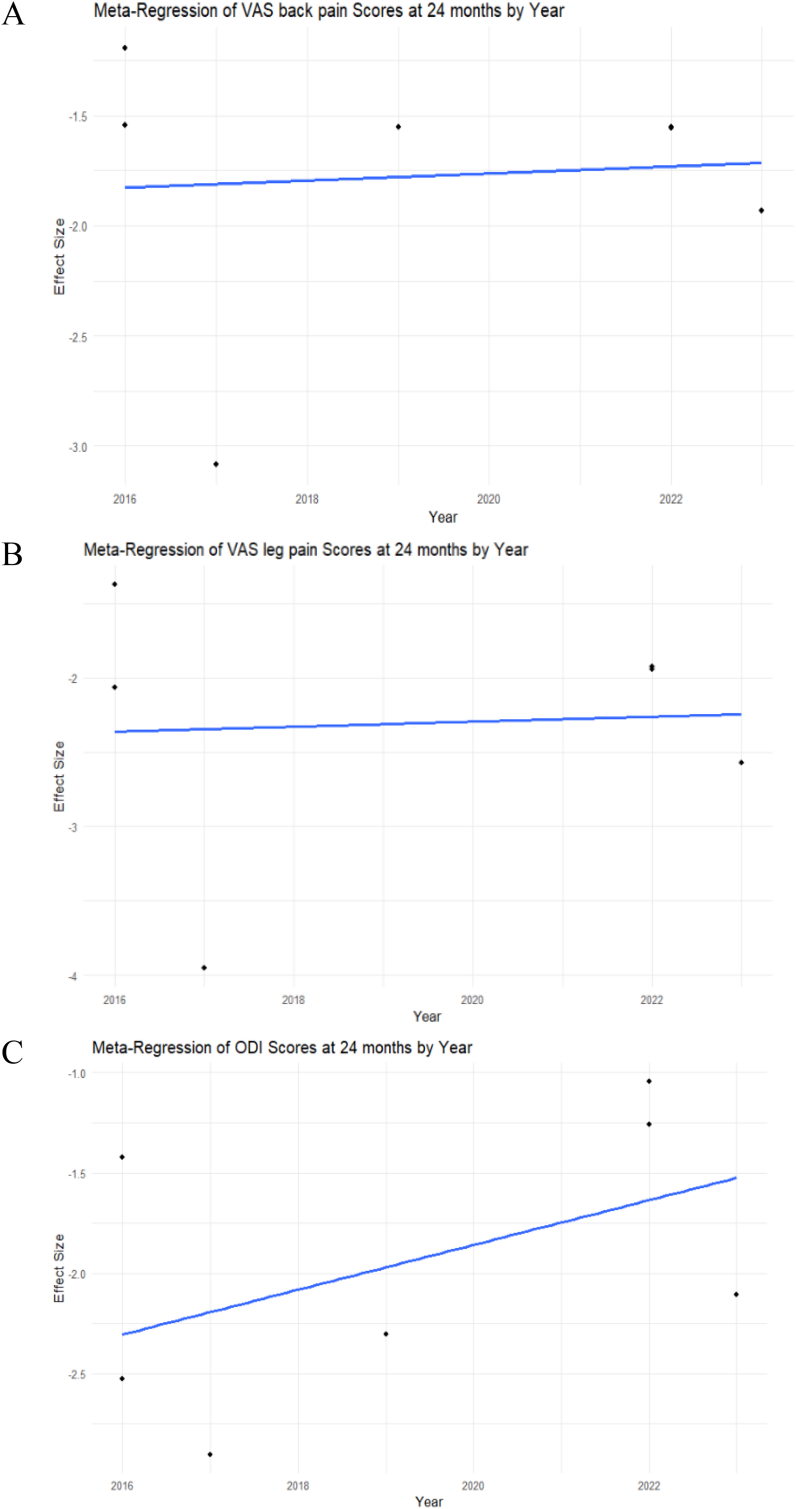
Meta-regression analysis of the VAS back and leg pain, ODI scores in MIS-TLIF patients. **(A)** VAS back pain score; **(B)** VAS leg pain score; **(C)** ODI score dynamics at 24 months post-operative in MIS-TLIF patients.

A meta-regression by year was performed for each study to assess heterogeneity. In the analysis of VAS back pain at 24 months, meta-regression showed statistical insignificance (intercept *p*-value = 0.8415), indicating that the examined factor did not have a significant impact on changes in the VAS back pain score in this model (Figure 6A).

The meta-regression analysis of VAS leg pain at 24 months demonstrated statistical insignificance (intercept *p*-value = 0.8984), indicating that the examined factor did not have a significant impact on changes in the VAS leg pain score in this model (Figure 6B).

The meta-regression analysis of ODI at 24 months demonstrated statistical insignificance (intercept *p*-value = 0.2069), indicating that the examined factor did not have a significant impact on changes in the ODI score in this model (Figure 6C).

### Publication bias assessment

**Figure 7:**
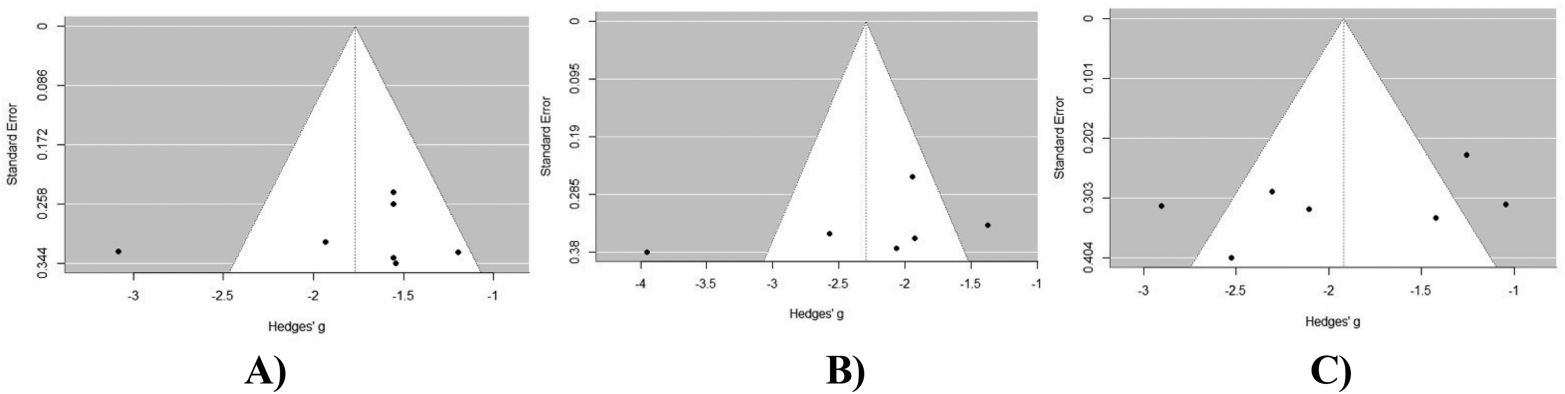
Publication bias assessment of outcomes at 24 months post-operatively in MIS-TLIF patients: **(A)** VAS back pain score; **(B)** VAS leg pain score; **(C)** ODI score dynamics at 12 months post-operative in MIS-TLIF patients.

Visual inspection of the VAS back pain score funnel plot (Figure 7A) suggests an asymmetric distribution of study results around the estimated effect size. This finding was further confirmed by Egger's test for publication bias, which yielded non-significant results (*p* > 0.05). A visual inspection of the VAS leg pain score funnel plot (Figure 7B) suggests a symmetric distribution of study results around the estimated effect size. This finding was further confirmed by Egger's test for publication bias, which yielded non-significant results (*p* > 0.05). Upon visual inspection of the ODI score funnel plot (Figure 7C) no asymmetry is evident, suggesting a symmetric distribution of study results around the estimated effect size. This finding was further confirmed by Egger's test for publication bias, which yielded non-significant results (*p* > 0.05).

## Discussion

This systematic review and meta-analysis aimed to evaluate the long-term clinical outcomes of MIS-TLIF surgery in the geriatric population with lumbar spondylolisthesis. Our analysis included seven studies published between 2015 and 2024, comprising a total of 280 geriatric patients with lumbar degenerative spondylolisthesis. The mean age of the participants ranged from 66.09–78.18 years. The primary outcomes of interest included VAS scores for back and leg pain, as well as ODI score changes at 12 and 24 months post-operatively. The pooled SMD across five groups for VAS back pain scores at 12 months post-operatively in geriatric MIS-TLIF patients was −4.30 [95% CI (−10.02; 1.42)]. However, by 24 months post-operatively, the VAS back pain improvement had decreased to an SMD of −1.77 [95% CI (−2.33, −1.21)]. For VAS leg pain scores, the pooled SMD from three groups showed an improvement of −2.46 [95% CI (−5.61; 0.68)] at 12 months, which remained relatively stable at 24 months with an SMD of −2.29 [95% CI (−3.22, −1.37)]. Similarly, the pooled SMD for ODI scores across five groups showed a slight improvement of −3.01 [95% CI (−6.02; −0.01)] at 12 months, which remained relatively consistent at 24 months, with an SMD of −1.92 [95% CI (−2.57, −1.27)].

Our findings align with the principles of Enhanced Recovery After Surgery (ERAS), which emphasize minimizing perioperative stress and accelerating functional recovery. As highlighted by Zaed et al. in their systematic review, MIS-TLIF is particularly suited to ERAS protocols due to its minimally invasive nature, which reduces tissue trauma, postoperative pain, and reliance on analgesia—critical factors in elderly patients with reduced physiological reserve (25–27). These advantages may promote faster return to mobility. Future integration of ERAS pathways tailored to MIS-TLIF could optimize outcomes in elderly patients, balancing cost-effectiveness with enhanced recovery.

Our findings significantly contribute to the existing body of evidence on post-operative outcomes of MIS-TLIF in geriatric patients. In his systematic review, Jonathan Huang (10) highlights substantial improvements in VAS back pain [−3.87, 95% CI (−4.97; −2.77)], VAS leg pain [−5.11, 95% CI (−6.69; −3.53)], and ODI [−30.70, 95% CI (−41.84; −19.55)] at six months post-operatively for geriatric patients undergoing MIS-TLIF. However, based on the results of our meta-analysis, by 24 months post-operatively, the magnitude of improvement decreases across all three primary outcomes, with back pain showing the least improvement. The elevation in VAS back and leg pain scores and in ODI scores at 24 months postoperatively among geriatric patients may stem from a confluence of factors, including disease progression, surgical complications, declining functional capacity, psychological variables, physiological age-related degeneration and homeostenosis.

Not surprisingly, elderly patients present unique challenges distinct from younger cohorts. Current approaches and techniques for managing spondylolisthesis in this demographic vary significantly. This systematic review examines the application of MIS-TLIF utilizing tubular systems, incorporating nerve root decompression and stabilization. In cases of spondylolisthesis among elderly patients, some have opted for endoscopic nerve root decompression. Nevertheless, contention exists among experts who argue that uncomplicated decompression may heighten the risk of reoperation due to destabilization, stemming from the removal of the osseous-musculotendinous complex and other posterior bony structures. The necessity of instrumented fusion in elderly patients with concurrent ailments warrants critical reevaluation, as the literature on this topic and its cost-effectiveness is contradicting. The cost-effectiveness analyses by Cheng and colleagues underscore that straightforward decompression proves less economically burdensome than supplementary fusion procedures (28). In another article, I.M. Austevoll (29) with coauthors emphasizes that open decompression without stabilization in elderly patients showed less improvement in the Physical Component Summary Score of the Medical Outcomes Study and a higher rate of reoperations compared to the fusion group. Orthopedic surgeons believe that displacement and dynamic instability in spondylolisthesis are best treated with stabilization surgery. Singh et al. (30) conducted a financial analysis of the total direct hospitalization costs (including blood, imaging, implants, physical or occupational therapy, and hospital stay) for 33 patients undergoing MIS-TLIF and 33 patients undergoing open TLIF, demonstrating that MIS-TLIF was less expensive than open TLIF (31).

When discussing postoperative complications in elderly patients with spondylolisthesis, the systematic review by Jin-Young Lee (32) emphasized the categorization of these complications into major and minor groups. The authors meticulously documented all postoperative complications among elderly patients in tabular format. Major complications listed in the table included wound infection, pulmonary embolism, and pneumonia, while minor complications encompassed urinary tract infection, anemia, dural tear, and delirium. Carreon et al. (33) highlight that 21% of elderly patients encountered at least one major complication, while 70% of them experienced at least one minor complication. The study also found that 49% of patients over 75 years old experienced a major complication. Shabat et al. (34) demonstrated that 52% of elderly patients experienced long-term complications after decompressive surgeries. Although this was not the primary focus of the current study, the authors of this meta-analysis recognize the importance of evaluating long-term outcomes after MIS-TLIF and incorporating these findings into comprehensive patient management strategies. Future studies should focus on the meta-analysis of the long-term safety outcomes of the MIS-TLIF in geriatric patients to provide the best evidence.

MIS-TLIF could become more cost-effective as more procedures are performed on an outpatient basis, helping to avoid high hospitalization costs (35). Parker et al. (2014) reported in their ICER (Incremental cost-effectiveness ratio) analysis that MIS-TLIF was more cost-effective than open TLIF over a two-year postoperative period. The total cost of MIS-TLIF (including direct and indirect expenses) was significantly lower −$38,563 compared to $47,858 for open TLIF (*P* = 0.03). A shorter hospital stay reduced direct costs by $1,758, while an earlier return to work lowered indirect costs by $8,474 (36, 37).

Dennis Chen Heath (38) emphasizes that further advancement of robot-assisted techniques may expand their application in complex cases. Both robot-assisted and CT-based navigation enable precise screw placement and a high level of safety in MIS-TLIF. As robotic technologies continue to evolve, they demonstrate more satisfactory results.

Based on our findings, several clinical recommendations can be made to guide surgeons in decision-making when managing geriatric patients with lumbar degenerative spondylolisthesis. The data from our meta-analysis indicate that MIS-TLIF offers significant short-term benefits, including reduced back and leg pain, and improved functional status. Given these advantages, MIS-TLIF should be considered the preferred approach for carefully selected elderly patients, especially those with comorbid conditions that increase surgical risk. Surgeons are encouraged to adopt Enhanced Recovery After Surgery (ERAS) protocols tailored to MIS-TLIF to optimize postoperative outcomes and reduce complication rates. However, the diminishing magnitude of improvement in pain and disability scores by 24 months postoperatively highlights the need for close long-term follow-up and proactive management of disease progression and age-related functional decline. Integrating multidisciplinary care, including physical therapy and geriatric rehabilitation, may help maintain functional gains and reduce the risk of reoperation. Future research should focus on refining patient selection criteria, improving surgical techniques through technological advancements like robotics and navigation systems, and assessing long-term safety and patient-reported outcomes to ensure evidence-based clinical practice.

Despite the comprehensive nature of the present systematic review and meta-analysis, several limitations should be acknowledged. Firstly, the review included only studies published in English, which may have introduced language bias and limited the generalizability of the findings. Secondly, the relatively small number of studies (seven) included in the meta-analysis raises concerns about the robustness and reliability of the pooled estimates. Although the studies adhered to a range of methodological standards, variations in study designs, and populations may have contributed to the observed high heterogeneity across the primary outcomes. Another notable limitation is that the included studies predominantly represent Asian populations, which may limit the generalizability of our findings to other regions with different demographic, genetic, and clinical characteristics. Caution should be exercised when applying these results to non-Asian populations, as differences in surgical practices, healthcare systems, and patient profiles may influence outcomes. Moreover, the assessment of publication bias revealed significant asymmetry in some of the funnel plots, indicating the potential for selective reporting of results that may distort the overall findings. One notable limitation is the absence of a detailed analysis of postoperative complications and patient-reported outcome measures. Evaluating both short-term and long-term complications and patient-reported outcome measures are essential for providing a more comprehensive risk-benefit assessment of MIS-TLIF in geriatric patients. While the primary focus of our study was on clinical outcomes such as VAS and ODI scores, future research should aim to systematically assess postoperative complications and patient-reported outcome measures to improve patient management strategies and guide clinical decision-making.

## Conclusion

In conclusion, our systematic review and meta-analysis provide compelling evidence supporting the long-term efficacy of MIS-TLIF for managing lumbar spondylolisthesis in geriatric patients. The findings suggest that MIS-TLIF is associated with significant reductions in back and leg pain, as well as improvements in disability scores over 12 months post-operatively. However, these improvements in pain and functional disability decline at 24 months postoperatively, which could be explained by the physiological nature of degenerative changes in the geriatric population. Future studies should focus on the meta-analysis of the long-term safety outcomes of the MIS-TLIF in geriatric patients to provide the best evidence.

## Data Availability

The datasets presented in this study can be found in online repositories. The names of the repository/repositories and accession number(s) can be found in the article/Supplementary Material.
