# Supplementary material for: Long term clinical outcomes of minimally invasive transforaminal interbody fusion (MIS-TLIF) for lumbar spondylolisthesis in a geriatric (>65 years) population: a systematic review and meta-analysis
Source: Front Surg. 2025 Mar 21;12:1517947. doi: 10.3389/fsurg.2025.1517947 (PMC11968762; doi:10.3389/fsurg.2025.1517947)
Supplement: Supplementary file 1 [file Table1.docx]

**Search strategy - Long Term Clinical Outcomes of Minimally Invasive Transforaminal Interbody Fusion (MIS-TLIF) for lumbar Spondylolisthesis in a Geriatric Population: a Systematic Review and Meta-Analysis.**

| **Pubmed** | **Web of Science** | **SCOPUS** | **Google Scholar** |
| --- | --- | --- | --- |
| Criteria: Search in all fields.  Filter: “Article type – books and documents, clinical trial, Randomized Controlled Trial”  Limits: 2015 and up to the present time.  1.“ MIS-TLIF and spondylolisthesis”- 7.  2. “Minimally invasive and spondylolisthesis”– 20.  3. “elderly and spondylolisthesis and fusion” – 71.  4. “fusion and spondylolisthesis“ - 89 | Criteria: Search in all fields.  Limits: 2015 and up to the present time.   1. “Geriatric and MIS-TLIF” – 2. 2. “elderly and spondylolisthesis and MIS-TLIF” -2. 3. “elderly spondylolisthesis and fusion” – 99. 4. “Minimally invasive transforaminal interbody fusion and elderly” – 29. | Criteria: Search in all fields.  Filter: “Language- English”  Limits: 2015 and up to the present time.   1. “MIS TLIF” – 655. 2. " Geriatric and MIS-TLIF– 12. 3. "Minimally invasive transforaminal interbody fusion and elderly" – 308. 4. "elderly and spondylolisthesis and MIS-TLIF" – 73. | Criteria: Search in all fields.  Filter: “in the title of the article”  Limits: 2015 and up to the present time.   1. "Minimally invasive transforaminal interbody fusion and elderly" **– 4.** 2. " Elderly AND spondylolisthesis AND fusion– 7860. 3. “Minimally invasive and spondylolisthesis” – 110. 4. elderly and spondylolisthesis -20. |

Начало формы

Конец формы

Impact of degenerative spinal diseases on bone mineral density of the lumbar spine in elderly women
